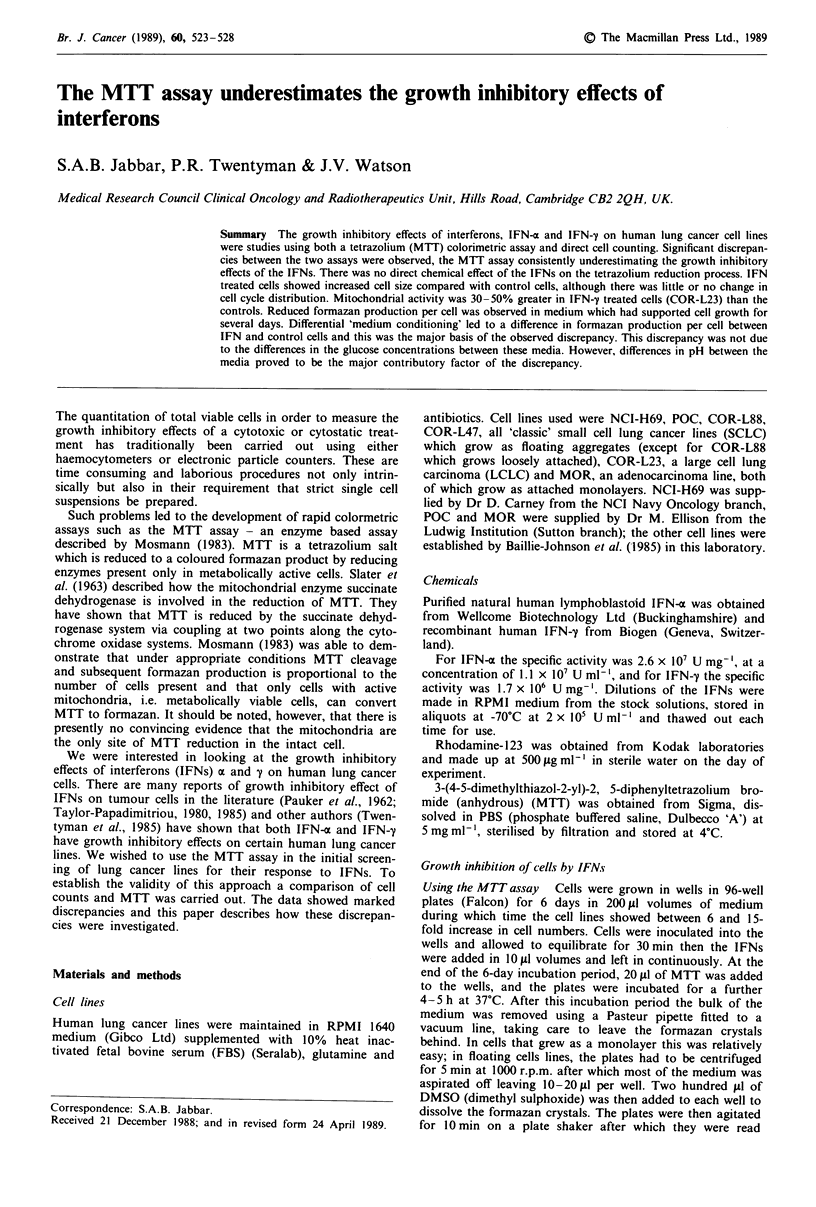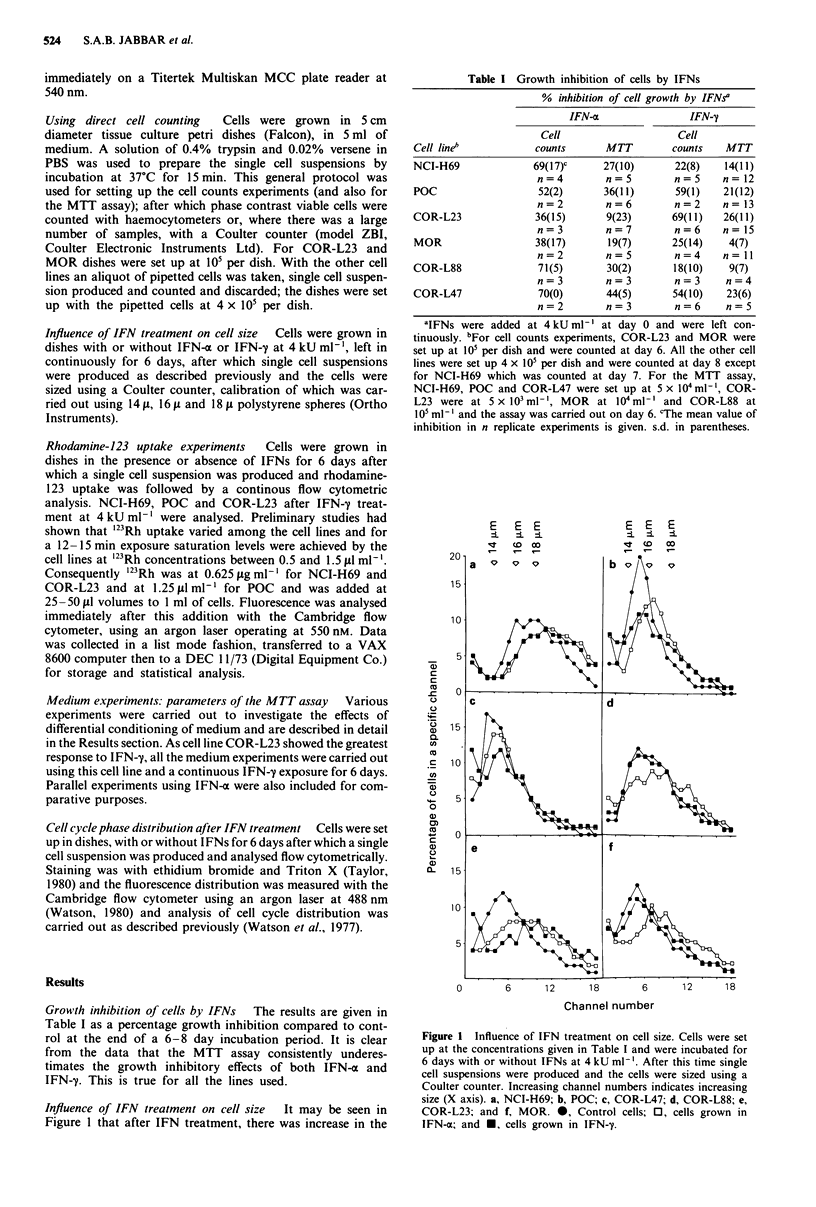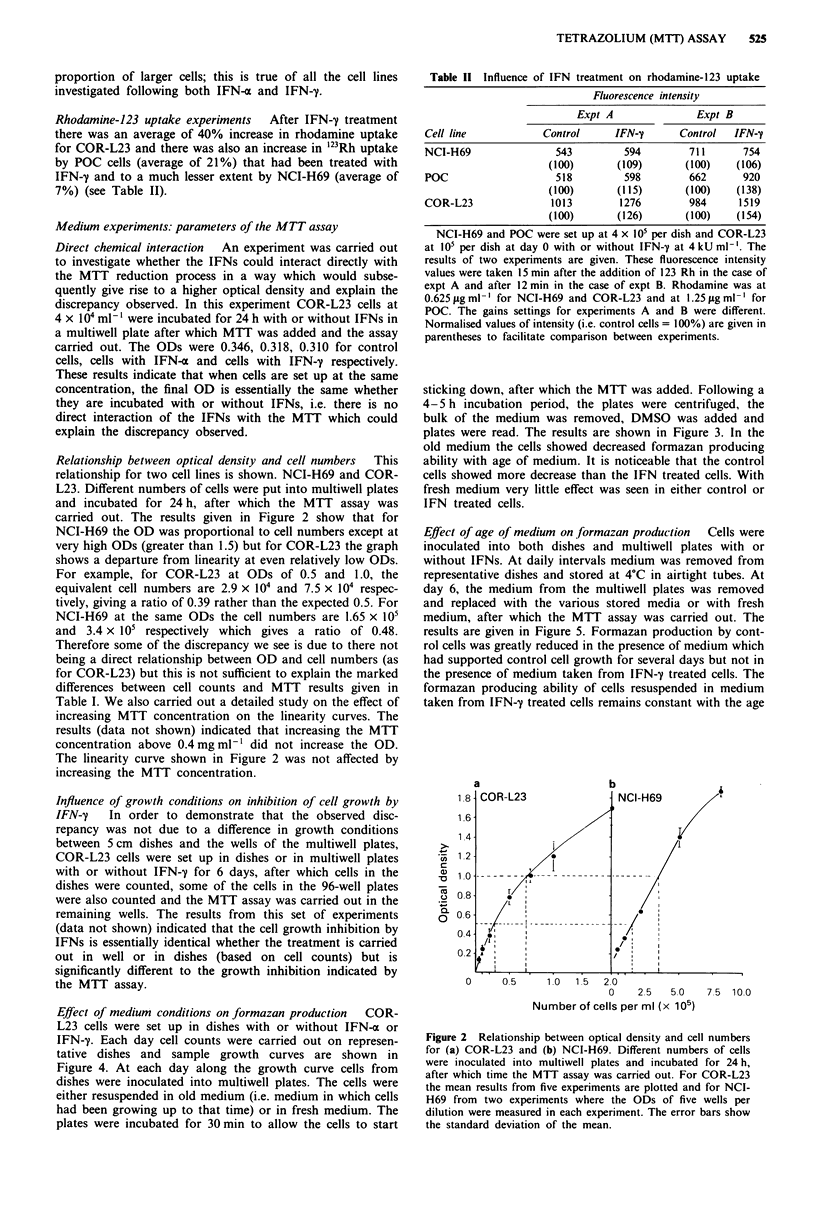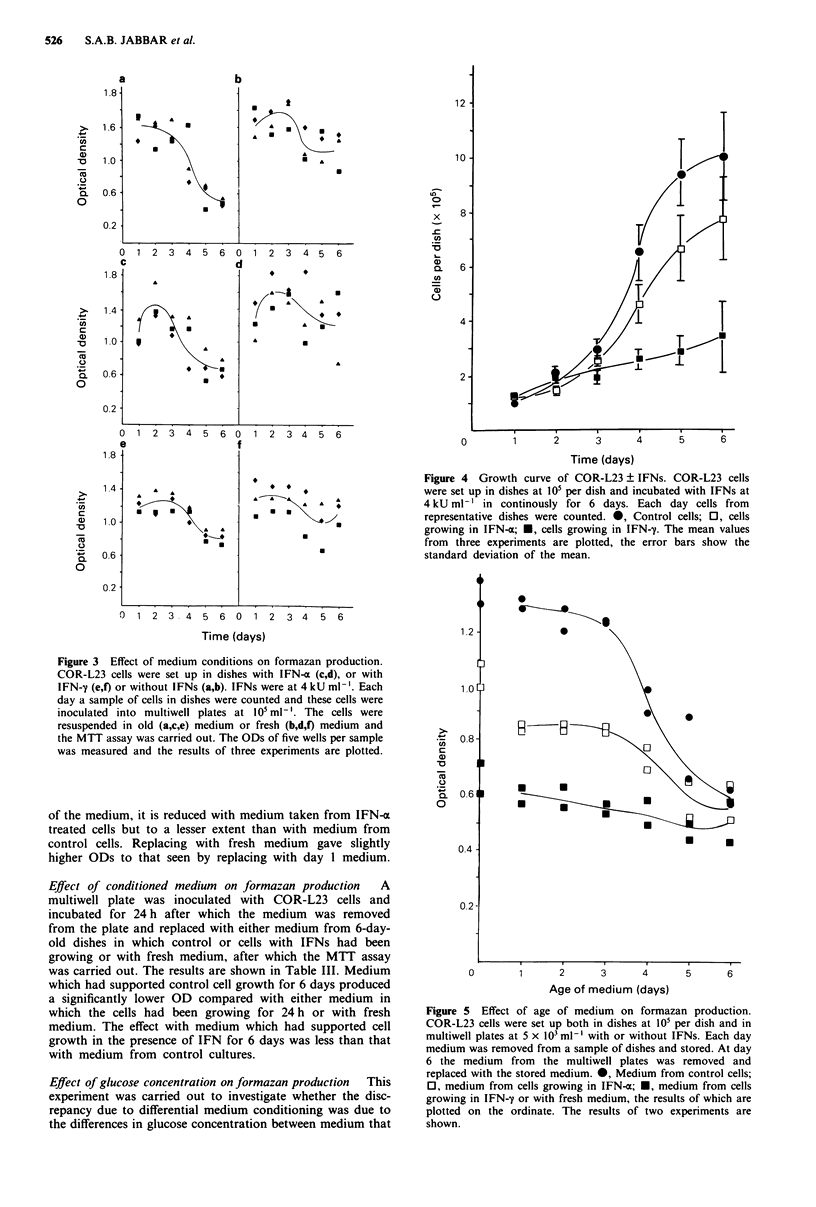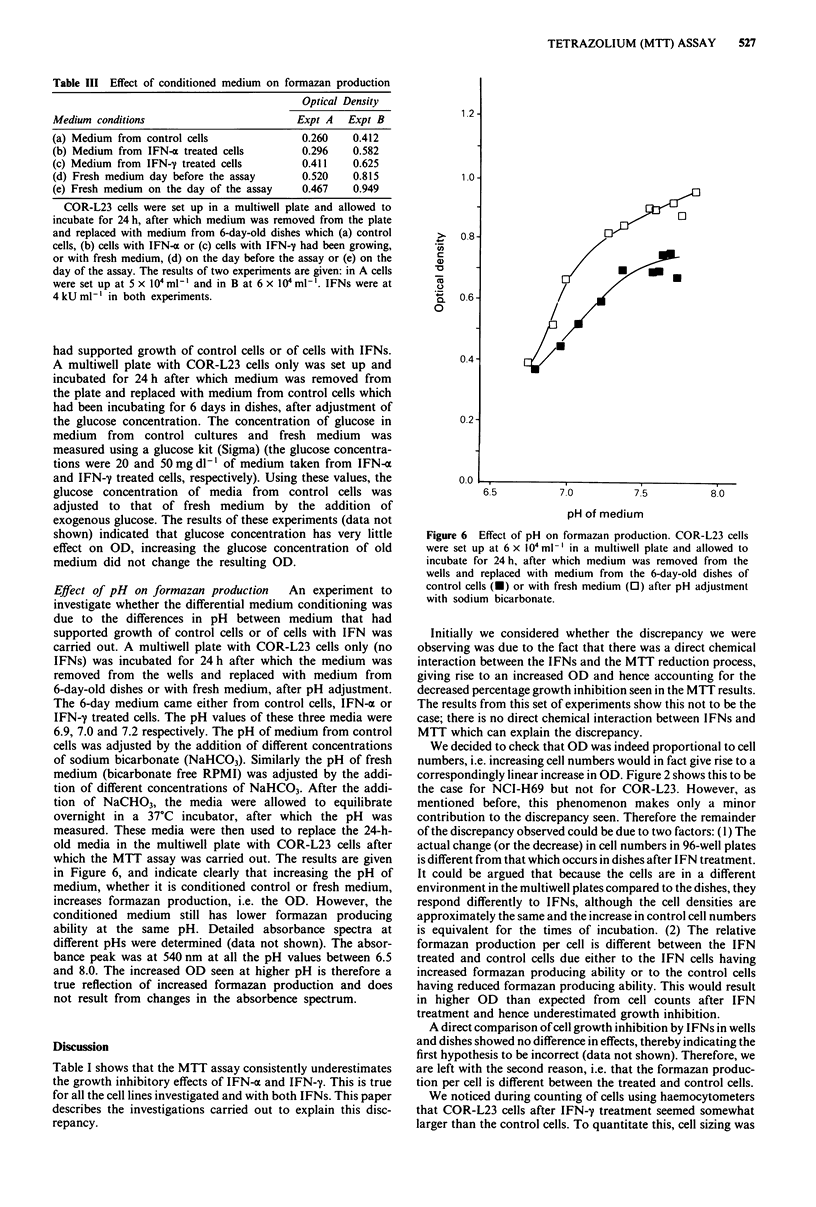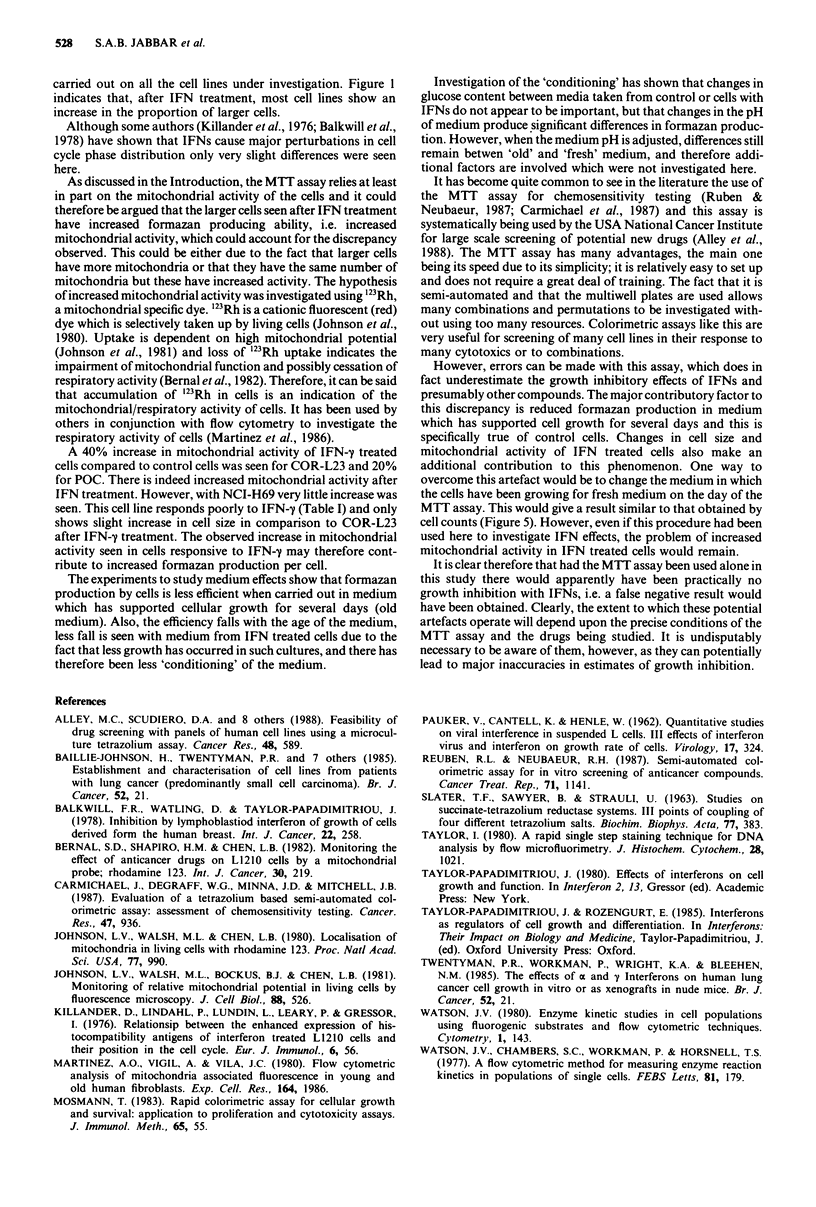# The MTT assay underestimates the growth inhibitory effects of interferons.

**DOI:** 10.1038/bjc.1989.306

**Published:** 1989-10

**Authors:** S. A. Jabbar, P. R. Twentyman, J. V. Watson

**Affiliations:** Medical Research Council Clinical Oncology, Cambridge, UK.

## Abstract

The growth inhibitory effects of interferons, IFN-alpha and IFN-gamma on human lung cancer cell lines were studied using both a tetrazolium (MTT) colorimetric assay and direct cell counting. Significant discrepancies between the two assays were observed, the MTT assay consistently underestimating the growth inhibitory effects of the IFNs. There was no direct chemical effect of the IFNs on the tetrazolium reduction process. IFN treated cells showed increased cell size compared with control cells, although there was little or no change in cell cycle distribution. Mitochondrial activity was 30-50% greater in IFN-gamma treated cells (COR-L23) than the controls. Reduced formazan production per cell was observed in medium which had supported cell growth for several days. Differential 'medium conditioning' led to a difference in formazan production per cell between IFN and control cells and this was the major basis of the observed discrepancy. This discrepancy was not due to the differences in the glucose concentrations between these media. However, differences in pH between the media proved to be the major contributory factor of the discrepancy.


Br. J. Cancer (1989), 60, 523-528 © The Macmillan Press Ltd., 1989

### The MTT assay underestimates the growth inhibitory effects of interferons

S.A.B. Jabbar, P.R. Twentyman & J.V. Watson

*Medical Research Council Clinical Oncology and Radiotherapeutics Unit, Hills Road, Cambridge CB2 2QH, UK.*

**Summary** The growth inhibitory effects of interferons, IFN-α and IFN-γ on human lung cancer cell lines were studies using both a tetrazolium (MTT) colorimetric assay and direct cell counting. Significant discrepancies between the two assays were observed, the MTT assay consistently underestimating the growth inhibitory effects of the IFNs. There was no direct chemical effect of the IFNs on the tetrazolium reduction process. IFN treated cells showed increased cell size compared with control cells, although there was little or no change in cell cycle distribution. Mitochondrial activity was 30-50% greater in IFN-γ treated cells (COR-L23) than the controls. Reduced formazan production per cell was observed in medium which had supported cell growth for several days. Differential 'medium conditioning' led to a difference in formazan production per cell between IFN and control cells and this was the major basis of the observed discrepancy. This discrepancy was not due to the differences in the glucose concentrations between these media. However, differences in pH between the media proved to be the major contributory factor of the discrepancy.

The quantitation of total viable cells in order to measure the growth inhibitory effects of a cytotoxic or cytostatic treatment has traditionally been carried out using either haemocytometers or electronic particle counters. These are time consuming and laborious procedures not only intrinsically but also in their requirement that strict single cell suspensions be prepared.

Such problems led to the development of rapid colormetric assays such as the MTT assay – an enzyme based assay described by Mosmann (1983). MTT is a tetrazolium salt which is reduced to a coloured formazan product by reducing enzymes present only in metabolically active cells. Slater *et al.* (1963) described how the mitochondrial enzyme succinate dehydrogenase is involved in the reduction of MTT. They have shown that MTT is reduced by the succinate dehydrogenase system via coupling at two points along the cytochrome oxidase systems. Mosmann (1983) was able to demonstrate that under appropriate conditions MTT cleavage and subsequent formazan production is proportional to the number of cells present and that only cells with active mitochondria, i.e. metabolically viable cells, can convert MTT to formazan. It should be noted, however, that there is presently no convincing evidence that the mitochondria are the only site of MTT reduction in the intact cell.

We were interested in looking at the growth inhibitory effects of interferons (IFNs) α and γ on human lung cancer cells. There are many reports of growth inhibitory effect of IFNs on tumour cells in the literature (Pauker *et al.*, 1962; Taylor-Papadimitriou, 1980, 1985) and other authors (Twentyman *et al.*, 1985) have shown that both IFN-α and IFN-γ have growth inhibitory effects on certain human lung cancer lines. We wished to use the MTT assay in the initial screening of lung cancer lines for their response to IFNs. To establish the validity of this approach a comparison of cell counts and MTT was carried out. The data showed marked discrepancies and this paper describes how these discrepancies were investigated.

### Materials and methods

### *Cell lines*

Human lung cancer lines were maintained in RPMI 1640 medium (Gibco Ltd) supplemented with 10% heat inactivated fetal bovine serum (FBS) (Seralab), glutamine and antibiotics. Cell lines used were NCI-H69, POC, COR-L88, COR-L47, all 'classic' small cell lung cancer lines (SCLC) which grow as floating aggregates (except for COR-L88 which grows loosely attached), COR-L23, a large cell lung carcinoma (LCLC) and MOR, an adenocarcinoma line, both of which grow as attached monolayers. NCI-H69 was supplied by Dr D. Carney from the NCI Navy Oncology branch, POC and MOR were supplied by Dr M. Ellison from the Ludwig Institution (Sutton branch); the other cell lines were established by Baillie-Johnson *et al.* (1985) in this laboratory.

### *Chemicals*

Purified natural human lymphoblastoid IFN-α was obtained from Wellcome Biotechnology Ltd (Buckinghamshire) and recombinant human IFN-γ from Biogen (Geneva, Switzerland).

For IFN-α the specific activity was $2.6 \times 10^7$ U mg$^{-1}$, at a concentration of $1.1 \times 10^7$ U ml$^{-1}$, and for IFN-γ the specific activity was $1.7 \times 10^6$ U mg$^{-1}$. Dilutions of the IFNs were made in RPMI medium from the stock solutions, stored in aliquots at -70°C at $2 \times 10^5$ U ml$^{-1}$ and thawed out each time for use.

Rhodamine-123 was obtained from Kodak laboratories and made up at 500 μg ml$^{-1}$ in sterile water on the day of experiment.

3-(4-5-dimethylthiazol-2-yl)-2, 5-diphenyltetrazolium bromide (anhydrous) (MTT) was obtained from Sigma, dissolved in PBS (phosphate buffered saline, Dulbecco 'A') at 5 mg ml$^{-1}$, sterilised by filtration and stored at 4°C.

### *Growth inhibition of cells by IFNs*

*Using the MTT assay* Cells were grown in wells in 96-well plates (Falcon) for 6 days in 200 μl volumes of medium during which time the cell lines showed between 6 and 15-fold increase in cell numbers. Cells were inoculated into the wells and allowed to equilibrate for 30 min then the IFNs were added in 10 μl volumes and left in continuously. At the end of the 6-day incubation period, 20 μl of MTT was added to the wells, and the plates were incubated for a further 4-5 h at 37°C. After this incubation period the bulk of the medium was removed using a Pasteur pipette fitted to a vacuum line, taking care to leave the formazan crystals behind. In cells that grew as a monolayer this was relatively easy; in floating cells lines, the plates had to be centrifuged for 5 min at 1000 r.p.m. after which most of the medium was aspirated off leaving 10-20 μl per well. Two hundred μl of DMSO (dimethyl sulphoxide) was then added to each well to dissolve the formazan crystals. The plates were then agitated for 10 min on a plate shaker after which they were read

Correspondence: S.A.B. Jabbar.
Received 21 December 1988; and in revised form 24 April 1989.

immediately on a Titertek Multiskan MCC plate reader at 540 nm.

*Using direct cell counting* Cells were grown in 5 cm diameter tissue culture petri dishes (Falcon), in 5 ml of medium. A solution of 0.4% trypsin and 0.02% versene in PBS was used to prepare the single cell suspensions by incubation at 37°C for 15 min. This general protocol was used for setting up the cell counts experiments (and also for the MTT assay); after which phase contrast viable cells were counted with haemocytometers or, where there was a large number of samples, with a Coulter counter (model ZBI, Coulter Electronic Instruments Ltd). For COR-L23 and MOR dishes were set up at $10^5$ per dish. With the other cell lines an aliquot of pipetted cells was taken, single cell suspension produced and counted and discarded; the dishes were set up with the pipetted cells at $4 \times 10^5$ per dish.

*Influence of IFN treatment on cell size* Cells were grown in dishes with or without IFN-α or IFN-γ at 4 kU $ml^{-1}$, left in continuously for 6 days, after which single cell suspensions were produced as described previously and the cells were sized using a Coulter counter, calibration of which was carried out using 14 μ, 16 μ and 18 μ polystyrene spheres (Ortho Instruments).

*Rhodamine-123 uptake experiments* Cells were grown in dishes in the presence or absence of IFNs for 6 days after which a single cell suspension was produced and rhodamine-123 uptake was followed by a continous flow cytometric analysis. NCI-H69, POC and COR-L23 after IFN-γ treatment at 4 kU $ml^{-1}$ were analysed. Preliminary studies had shown that $^{123}$Rh uptake varied among the cell lines and for a 12–15 min exposure saturation levels were achieved by the cell lines at $^{123}$Rh concentrations between 0.5 and 1.5 μl $ml^{-1}$. Consequently $^{123}$Rh was at 0.625 μg $ml^{-1}$ for NCI-H69 and COR-L23 and at 1.25 μl $ml^{-1}$ for POC and was added at 25–50 μl volumes to 1 ml of cells. Fluorescence was analysed immediately after this addition with the Cambridge flow cytometer, using an argon laser operating at 550 nM. Data was collected in a list mode fashion, transferred to a VAX 8600 computer then to a DEC 11/73 (Digital Equipment Co.) for storage and statistical analysis.

*Medium experiments: parameters of the MTT assay* Various experiments were carried out to investigate the effects of differential conditioning of medium and are described in detail in the Results section. As cell line COR-L23 showed the greatest response to IFN-γ, all the medium experiments were carried out using this cell line and a continuous IFN-γ exposure for 6 days. Parallel experiments using IFN-α were also included for comparative purposes.

*Cell cycle phase distribution after IFN treatment* Cells were set up in dishes, with or without IFNs for 6 days after which a single cell suspension was produced and analysed flow cytometrically. Staining was with ethidium bromide and Triton X (Taylor, 1980) and the fluorescence distribution was measured with the Cambridge flow cytometer using an argon laser at 488 nm (Watson, 1980) and analysis of cell cycle distribution was carried out as described previously (Watson *et al.*, 1977).

### Results

*Growth inhibition of cells by IFNs* The results are given in Table I as a percentage growth inhibition compared to control at the end of a 6–8 day incubation period. It is clear from the data that the MTT assay consistently underestimates the growth inhibitory effects of both IFN-α and IFN-γ. This is true for all the lines used.

*Influence of IFN treatment on cell size* It may be seen in Figure 1 that after IFN treatment, there was increase in the

**Table I** Growth inhibition of cells by IFNs

| | *% inhibition of cell growth by IFNs*[a] | | | |
|---|---|---|---|---|
| | *IFN-α* | | *IFN-γ* | |
| *Cell line*[b] | *Cell counts* | *MTT* | *Cell counts* | *MTT* |
| NCI-H69 | 69(17)[c] | 27(10) | 22(8) | 14(11) |
| | $n = 4$ | $n = 5$ | $n = 5$ | $n = 12$ |
| POC | 52(2) | 36(11) | 59(1) | 21(12) |
| | $n = 2$ | $n = 6$ | $n = 2$ | $n = 13$ |
| COR-L23 | 36(15) | 9(23) | 69(11) | 26(11) |
| | $n = 3$ | $n = 7$ | $n = 6$ | $n = 15$ |
| MOR | 38(17) | 19(7) | 25(14) | 4(7) |
| | $n = 2$ | $n = 5$ | $n = 4$ | $n = 11$ |
| COR-L88 | 71(5) | 30(2) | 18(10) | 9(7) |
| | $n = 3$ | $n = 3$ | $n = 3$ | $n = 4$ |
| COR-L47 | 70(0) | 44(5) | 54(10) | 23(6) |
| | $n = 2$ | $n = 3$ | $n = 6$ | $n = 5$ |

[a]IFNs were added at 4 kU $ml^{-1}$ at day 0 and were left continuously. [b]For cell counts experiments, COR-L23 and MOR were set up at $10^5$ per dish and were counted at day 6. All the other cell lines were set up $4 \times 10^5$ per dish and were counted at day 8 except for NCI-H69 which was counted at day 7. For the MTT assay, NCI-H69, POC and COR-L47 were set up at $5 \times 10^4$ $ml^{-1}$, COR-L23 were at $5 \times 10^3$ $ml^{-1}$, MOR at $10^4$ $ml^{-1}$ and COR-L88 at $10^5$ $ml^{-1}$ and the assay was carried out on day 6. [c]The mean value of inhibition in $n$ replicate experiments is given. s.d. in parentheses.

**Figure 1** Influence of IFN treatment on cell size. Cells were set up at the concentrations given in Table I and were incubated for 6 days with or without IFNs at 4 kU $ml^{-1}$. After this time single cell suspensions were produced and the cells were sized using a Coulter counter. Increasing channel numbers indicates increasing size (X axis). **a**, NCI-H69; **b**, POC; **c**, COR-L47; **d**, COR-L88; **e**, COR-L23; and **f**, MOR. ●, Control cells; □, cells grown in IFN-α; and ■, cells grown in IFN-γ.

proportion of larger cells; this is true of all the cell lines investigated following both IFN-α and IFN-γ.

*Rhodamine-123 uptake experiments* After IFN-γ treatment there was an average of 40% increase in rhodamine uptake for COR-L23 and there was also an increase in $^{123}$Rh uptake by POC cells (average of 21%) that had been treated with IFN-γ and to a much lesser extent by NCI-H69 (average of 7%) (see Table II).

*Medium experiments: parameters of the MTT assay*

*Direct chemical interaction* An experiment was carried out to investigate whether the IFNs could interact directly with the MTT reduction process in a way which would subsequently give rise to a higher optical density and explain the discrepancy observed. In this experiment COR-L23 cells at $4 \times 10^4$ ml$^{-1}$ were incubated for 24 h with or without IFNs in a multiwell plate after which MTT was added and the assay carried out. The ODs were 0.346, 0.318, 0.310 for control cells, cells with IFN-α and cells with IFN-γ respectively. These results indicate that when cells are set up at the same concentration, the final OD is essentially the same whether they are incubated with or without IFNs, i.e. there is no direct interaction of the IFNs with the MTT which could explain the discrepancy observed.

*Relationship between optical density and cell numbers* This relationship for two cell lines is shown. NCI-H69 and COR-L23. Different numbers of cells were put into multiwell plates and incubated for 24 h, after which the MTT assay was carried out. The results given in Figure 2 show that for NCI-H69 the OD was proportional to cell numbers except at very high ODs (greater than 1.5) but for COR-L23 the graph shows a departure from linearity at even relatively low ODs. For example, for COR-L23 at ODs of 0.5 and 1.0, the equivalent cell numbers are $2.9 \times 10^4$ and $7.5 \times 10^4$ respectively, giving a ratio of 0.39 rather than the expected 0.5. For NCI-H69 at the same ODs the cell numbers are $1.65 \times 10^5$ and $3.4 \times 10^5$ respectively which gives a ratio of 0.48. Therefore some of the discrepancy we see is due to there not being a direct relationship between OD and cell numbers (as for COR-L23) but this is not sufficient to explain the marked differences between cell counts and MTT results given in Table I. We also carried out a detailed study on the effect of increasing MTT concentration on the linearity curves. The results (data not shown) indicated that increasing the MTT concentration above 0.4 mg ml$^{-1}$ did not increase the OD. The linearity curve shown in Figure 2 was not affected by increasing the MTT concentration.

*Influence of growth conditions on inhibition of cell growth by IFN-γ* In order to demonstrate that the observed discrepancy was not due to a difference in growth conditions between 5 cm dishes and the wells of the multiwell plates, COR-L23 cells were set up in dishes or in multiwell plates with or without IFN-γ for 6 days, after which cells in the dishes were counted, some of the cells in the 96-well plates were also counted and the MTT assay was carried out in the remaining wells. The results from this set of experiments (data not shown) indicated that the cell growth inhibition by IFNs is essentially identical whether the treatment is carried out in well or in dishes (based on cell counts) but is significantly different to the growth inhibition indicated by the MTT assay.

*Effect of medium conditions on formazan production* COR-L23 cells were set up in dishes with or without IFN-α or IFN-γ. Each day cell counts were carried out on representative dishes and sample growth curves are shown in Figure 4. At each day along the growth curve cells from dishes were inoculated into multiwell plates. The cells were either resuspended in old medium (i.e. medium in which cells had been growing up to that time) or in fresh medium. The plates were incubated for 30 min to allow the cells to start sticking down, after which the MTT was added. Following a 4–5 h incubation period, the plates were centrifuged, the bulk of the medium was removed, DMSO was added and plates were read. The results are shown in Figure 3. In the old medium the cells showed decreased formazan producing ability with age of medium. It is noticeable that the control cells showed more decrease than the IFN treated cells. With fresh medium very little effect was seen in either control or IFN treated cells.

**Table II** Influence of IFN treatment on rhodamine-123 uptake

| | *Fluorescence intensity* | | | |
|---|---|---|---|---|
| | *Expt A* | | *Expt B* | |
| *Cell line* | *Control* | *IFN-γ* | *Control* | *IFN-γ* |
| NCI-H69 | 543 | 594 | 711 | 754 |
| | (100) | (109) | (100) | (106) |
| POC | 518 | 598 | 662 | 920 |
| | (100) | (115) | (100) | (138) |
| COR-L23 | 1013 | 1276 | 984 | 1519 |
| | (100) | (126) | (100) | (154) |

NCI-H69 and POC were set up at $4 \times 10^5$ per dish and COR-L23 at $10^5$ per dish at day 0 with or without IFN-γ at 4 kU ml$^{-1}$. The results of two experiments are given. These fluorescence intensity values were taken 15 min after the addition of 123 Rh in the case of expt A and after 12 min in the case of expt B. Rhodamine was at 0.625 μg ml$^{-1}$ for NCI-H69 and COR-L23 and at 1.25 μg ml$^{-1}$ for POC. The gains settings for experiments A and B were different. Normalised values of intensity (i.e. control cells = 100%) are given in parentheses to facilitate comparison between experiments.

*Effect of age of medium on formazan production* Cells were inoculated into both dishes and multiwell plates with or without IFNs. At daily intervals medium was removed from representative dishes and stored at 4°C in airtight tubes. At day 6, the medium from the multiwell plates was removed and replaced with the various stored media or with fresh medium, after which the MTT assay was carried out. The results are given in Figure 5. Formazan production by control cells was greatly reduced in the presence of medium which had supported control cell growth for several days but not in the presence of medium taken from IFN-γ treated cells. The formazan producing ability of cells resuspended in medium taken from IFN-γ treated cells remains constant with the age

**Figure 2** Relationship between optical density and cell numbers for (**a**) COR-L23 and (**b**) NCI-H69. Different numbers of cells were inoculated into multiwell plates and incubated for 24 h, after which time the MTT assay was carried out. For COR-L23 the mean results from five experiments are plotted and for NCI-H69 from two experiments where the ODs of five wells per dilution were measured in each experiment. The error bars show the standard deviation of the mean.

**Figure 3** Effect of medium conditions on formazan production. COR-L23 cells were set up in dishes with IFN-α (**c**,**d**), or with IFN-γ (**e**,**f**) or without IFNs (**a**,**b**). IFNs were at 4 kU ml$^{-1}$. Each day a sample of cells in dishes were counted and these cells were inoculated into multiwell plates at $10^5$ ml$^{-1}$. The cells were resuspended in old (**a**,**c**,**e**) medium or fresh (**b**,**d**,**f**) medium and the MTT assay was carried out. The ODs of five wells per sample was measured and the results of three experiments are plotted.

of the medium, it is reduced with medium taken from IFN-α treated cells but to a lesser extent than with medium from control cells. Replacing with fresh medium gave slightly higher ODs to that seen by replacing with day 1 medium.

*Effect of conditioned medium on formazan production* A multiwell plate was inoculated with COR-L23 cells and incubated for 24 h after which the medium was removed from the plate and replaced with either medium from 6-day-old dishes in which control or cells with IFNs had been growing or with fresh medium, after which the MTT assay was carried out. The results are shown in Table III. Medium which had supported control cell growth for 6 days produced a significantly lower OD compared with either medium in which the cells had been growing for 24 h or with fresh medium. The effect with medium which had supported cell growth in the presence of IFN for 6 days was less than that with medium from control cultures.

*Effect of glucose concentration on formazan production* This experiment was carried out to investigate whether the discrepancy due to differential medium conditioning was due to the differences in glucose concentration between medium that

**Figure 4** Growth curve of COR-L23 ± IFNs. COR-L23 cells were set up in dishes at $10^5$ per dish and incubated with IFNs at 4 kU ml$^{-1}$ in continously for 6 days. Each day cells from representative dishes were counted. ●, Control cells; □, cells growing in IFN-α; ■, cells growing in IFN-γ. The mean values from three experiments are plotted, the error bars show the standard deviation of the mean.

**Figure 5** Effect of age of medium on formazan production. COR-L23 cells were set up both in dishes at $10^5$ per dish and in multiwell plates at $5 \times 10^3$ ml$^{-1}$ with or without IFNs. Each day medium was removed from a sample of dishes and stored. At day 6 the medium from the multiwell plates was removed and replaced with the stored medium. ●, Medium from control cells; □, medium from cells growing in IFN-α; ■, medium from cells growing in IFN-γ or with fresh medium, the results of which are plotted on the ordinate. The results of two experiments are shown.

**Table III** Effect of conditioned medium on formazan production

| Medium conditions | *Optical Density* Expt A | Expt B |
|---|---|---|
| (a) Medium from control cells | 0.260 | 0.412 |
| (b) Medium from IFN-α treated cells | 0.296 | 0.582 |
| (c) Medium from IFN-γ treated cells | 0.411 | 0.625 |
| (d) Fresh medium day before the assay | 0.520 | 0.815 |
| (e) Fresh medium on the day of the assay | 0.467 | 0.949 |

COR-L23 cells were set up in a multiwell plate and allowed to incubate for 24 h, after which medium was removed from the plate and replaced with medium from 6-day-old dishes which (a) control cells, (b) cells with IFN-α or (c) cells with IFN-γ had been growing, or with fresh medium, (d) on the day before the assay or (e) on the day of the assay. The results of two experiments are given: in A cells were set up at $5 \times 10^4 ml^{-1}$ and in B at $6 \times 10^4 ml^{-1}$. IFNs were at $4 kU ml^{-1}$ in both experiments.

had supported growth of control cells or of cells with IFNs. A multiwell plate with COR-L23 cells only was set up and incubated for 24 h after which medium was removed from the plate and replaced with medium from control cells which had been incubating for 6 days in dishes, after adjustment of the glucose concentration. The concentration of glucose in medium from control cultures and fresh medium was measured using a glucose kit (Sigma) (the glucose concentrations were 20 and $50 mg dl^{-1}$ of medium taken from IFN-α and IFN-γ treated cells, respectively). Using these values, the glucose concentration of media from control cells was adjusted to that of fresh medium by the addition of exogenous glucose. The results of these experiments (data not shown) indicated that glucose concentration has very little effect on OD, increasing the glucose concentration of old medium did not change the resulting OD.

*Effect of pH on formazan production* An experiment to investigate whether the differential medium conditioning was due to the differences in pH between medium that had supported growth of control cells or of cells with IFN was carried out. A multiwell plate with COR-L23 cells only (no IFNs) was incubated for 24 h after which the medium was removed from the wells and replaced with medium from 6-day-old dishes or with fresh medium, after pH adjustment. The 6-day medium came either from control cells, IFN-α or IFN-γ treated cells. The pH values of these three media were 6.9, 7.0 and 7.2 respectively. The pH of medium from control cells was adjusted by the addition of different concentrations of sodium bicarbonate ($NaHCO_3$). Similarly the pH of fresh medium (bicarbonate free RPMI) was adjusted by the addition of different concentrations of $NaHCO_3$. After the addition of $NaCHO_3$, the media were allowed to equilibrate overnight in a 37°C incubator, after which the pH was measured. These media were then used to replace the 24-h-old media in the multiwell plate with COR-L23 cells after which the MTT assay was carried out. The results are given in Figure 6, and indicate clearly that increasing the pH of medium, whether it is conditioned control or fresh medium, increases formazan production, i.e. the OD. However, the conditioned medium still has lower formazan producing ability at the same pH. Detailed absorbance spectra at different pHs were determined (data not shown). The absorbance peak was at 540 nm at all the pH values between 6.5 and 8.0. The increased OD seen at higher pH is therefore a true reflection of increased formazan production and does not result from changes in the absorbence spectrum.

### Discussion

Table I shows that the MTT assay consistently underestimates the growth inhibitory effects of IFN-α and IFN-γ. This is true for all the cell lines investigated and with both IFNs. This paper describes the investigations carried out to explain this discrepancy.

**Figure 6** Effect of pH on formazan production. COR-L23 cells were set up at $6 \times 10^4 ml^{-1}$ in a multiwell plate and allowed to incubate for 24 h, after which medium was removed from the wells and replaced with medium from the 6-day-old dishes of control cells (■) or with fresh medium (□) after pH adjustment with sodium bicarbonate.

Initially we considered whether the discrepancy we were observing was due to the fact that there was a direct chemical interaction between the IFNs and the MTT reduction process, giving rise to an increased OD and hence accounting for the decreased percentage growth inhibition seen in the MTT results. The results from this set of experiments show this not to be the case; there is no direct chemical interaction between IFNs and MTT which can explain the discrepancy.

We decided to check that OD was indeed proportional to cell numbers, i.e. increasing cell numbers would in fact give rise to a correspondingly linear increase in OD. Figure 2 shows this to be the case for NCI-H69 but not for COR-L23. However, as mentioned before, this phenomenon makes only a minor contribution to the discrepancy seen. Therefore the remainder of the discrepancy observed could be due to two factors: (1) The actual change (or the decrease) in cell numbers in 96-well plates is different from that which occurs in dishes after IFN treatment. It could be argued that because the cells are in a different environment in the multiwell plates compared to the dishes, they respond differently to IFNs, although the cell densities are approximately the same and the increase in control cell numbers is equivalent for the times of incubation. (2) The relative formazan production per cell is different between the IFN treated and control cells due either to the IFN cells having increased formazan producing ability or to the control cells having reduced formazan producing ability. This would result in higher OD than expected from cell counts after IFN treatment and hence underestimated growth inhibition.

A direct comparison of cell growth inhibition by IFNs in wells and dishes showed no difference in effects, thereby indicating the first hypothesis to be incorrect (data not shown). Therefore, we are left with the second reason, i.e. that the formazan production per cell is different between the treated and control cells.

We noticed during counting of cells using haemocytometers that COR-L23 cells after IFN-γ treatment seemed somewhat larger than the control cells. To quantitate this, cell sizing was carried out on all the cell lines under investigation. Figure 1 indicates that, after IFN treatment, most cell lines show an increase in the proportion of larger cells.

Although some authors (Killander *et al.*, 1976; Balkwill *et al.*, 1978) have shown that IFNs cause major perturbations in cell cycle phase distribution only very slight differences were seen here.

As discussed in the Introduction, the MTT assay relies at least in part on the mitochondrial activity of the cells and it could therefore be argued that the larger cells seen after IFN treatment have increased formazan producing ability, i.e. increased mitochondrial activity, which could account for the discrepancy observed. This could be either due to the fact that larger cells have more mitochondria or that they have the same number of mitochondria but these have increased activity. The hypothesis of increased mitochondrial activity was investigated using $^{123}$Rh, a mitochondrial specific dye. $^{123}$Rh is a cationic fluorescent (red) dye which is selectively taken up by living cells (Johnson *et al.*, 1980). Uptake is dependent on high mitochondrial potential (Johnson *et al.*, 1981) and loss of $^{123}$Rh uptake indicates the impairment of mitochondrial function and possibly cessation of respiratory activity (Bernal *et al.*, 1982). Therefore, it can be said that accumulation of $^{123}$Rh in cells is an indication of the mitochondrial/respiratory activity of cells. It has been used by others in conjunction with flow cytometry to investigate the respiratory activity of cells (Martinez *et al.*, 1986).

A 40% increase in mitochondrial activity of IFN-$\gamma$ treated cells compared to control cells was seen for COR-L23 and 20% for POC. There is indeed increased mitochondrial activity after IFN treatment. However, with NCI-H69 very little increase was seen. This cell line responds poorly to IFN-$\gamma$ (Table I) and only shows slight increase in cell size in comparison to COR-L23 after IFN-$\gamma$ treatment. The observed increase in mitochondrial activity seen in cells responsive to IFN-$\gamma$ may therefore contribute to increased formazan production per cell.

The experiments to study medium effects show that formazan production by cells is less efficient when carried out in medium which has supported cellular growth for several days (old medium). Also, the efficiency falls with the age of the medium, less fall is seen with medium from IFN treated cells due to the fact that less growth has occurred in such cultures, and there has therefore been less 'conditioning' of the medium.

Investigation of the 'conditioning' has shown that changes in glucose content between media taken from control or cells with IFNs do not appear to be important, but that changes in the pH of medium produce significant differences in formazan production. However, when the medium pH is adjusted, differences still remain betwen 'old' and 'fresh' medium, and therefore additional factors are involved which were not investigated here.

It has become quite common to see in the literature the use of the MTT assay for chemosensitivity testing (Ruben & Neubaeur, 1987; Carmichael *et al.*, 1987) and this assay is systematically being used by the USA National Cancer Institute for large scale screening of potential new drugs (Alley *et al.*, 1988). The MTT assay has many advantages, the main one being its speed due to its simplicity; it is relatively easy to set up and does not require a great deal of training. The fact that it is semi-automated and that the multiwell plates are used allows many combinations and permutations to be investigated without using too many resources. Colorimetric assays like this are very useful for screening of many cell lines in their response to many cytotoxics or to combinations.

However, errors can be made with this assay, which does in fact underestimate the growth inhibitory effects of IFNs and presumably other compounds. The major contributory factor to this discrepancy is reduced formazan production in medium which has supported cell growth for several days and this is specifically true of control cells. Changes in cell size and mitochondrial activity of IFN treated cells also make an additional contribution to this phenomenon. One way to overcome this artefact would be to change the medium in which the cells have been growing for fresh medium on the day of the MTT assay. This would give a result similar to that obtained by cell counts (Figure 5). However, even if this procedure had been used here to investigate IFN effects, the problem of increased mitochondrial activity in IFN treated cells would remain.

It is clear therefore that had the MTT assay been used alone in this study there would apparently have been practically no growth inhibition with IFNs, i.e. a false negative result would have been obtained. Clearly, the extent to which these potential artefacts operate will depend upon the precise conditions of the MTT assay and the drugs being studied. It is undisputably necessary to be aware of them, however, as they can potentially lead to major inaccuracies in estimates of growth inhibition.

### References

ALLEY, M.C., SCUDIERO, D.A. and 8 others (1988). Feasibility of drug screening with panels of human cell lines using a microculture tetrazolium assay. *Cancer Res.*, **48**, 589.

BAILLIE-JOHNSON, H., TWENTYMAN, P.R. and 7 others (1985). Establishment and characterisation of cell lines from patients with lung cancer (predominantly small cell carcinoma). *Br. J. Cancer*, **52**, 21.

BALKWILL, F.R., WATLING, D. & TAYLOR-PAPADIMITRIOU, J. (1978). Inhibition by lymphoblastiod interferon of growth of cells derived form the human breast. *Int. J. Cancer*, **22**, 258.

BERNAL, S.D., SHAPIRO, H.M. & CHEN, L.B. (1982). Monitoring the effect of anticancer drugs on L1210 cells by a mitochondrial probe; rhodamine 123. *Int. J. Cancer*, **30**, 219.

CARMICHAEL, J., DEGRAFF, W.G., MINNA, J.D. & MITCHELL, J.B. (1987). Evaluation of a tetrazolium based semi-automated colorimetric assay: assessment of chemosensitivity testing. *Cancer. Res.*, **47**, 936.

JOHNSON, L.V., WALSH, M.L. & CHEN, L.B. (1980). Localisation of mitochondria in living cells with rhodamine 123. *Proc. Natl Acad. Sci. USA*, **77**, 990.

JOHNSON, L.V., WALSH, M.L., BOCKUS, B.J. & CHEN, L.B. (1981). Monitoring of relative mitochondrial potential in living cells by fluorescence microscopy. *J. Cell Biol.*, **88**, 526.

KILLANDER, D., LINDAHL, P., LUNDIN, L., LEARY, P. & GRESSOR, I. (1976). Relationsip between the enhanced expression of histocompatibility antigens of interferon treated L1210 cells and their position in the cell cycle. *Eur. J. Immunol.*, **6**, 56.

MARTINEZ, A.O., VIGIL, A. & VILA, J.C. (1980). Flow cytometric analysis of mitochondria associated fluorescence in young and old human fibroblasts. *Exp. Cell. Res.*, **164**, 1986.

MOSMANN, T. (1983). Rapid colorimetric assay for cellular growth and survival: application to proliferation and cytotoxicity assays. *J. Immunol. Meth.*, **65**, 55.

PAUKER, V., CANTELL, K. & HENLE, W. (1962). Quantitative studies on viral interference in suspended L cells. III effects of interferon virus and interferon on growth rate of cells. *Virology*, **17**, 324.

REUBEN, R.L. & NEUBAEUR, R.H. (1987). Semi-automated colorimetric assay for in vitro screening of anticancer compounds. *Cancer Treat. Rep.*, **71**, 1141.

SLATER, T.F., SAWYER, B. & STRAULI, U. (1963). Studies on succinate-tetrazolium reductase systems. III points of coupling of four different tetrazolium salts. *Biochim. Biophys. Acta*, **77**, 383.

TAYLOR, I. (1980). A rapid single step staining technique for DNA analysis by flow microfluorimetry. *J. Histochem. Cytochem.*, **28**, 1021.

TAYLOR-PAPADIMITRIOU, J. (1980). Effects of interferons on cell growth and function. In *Interferon 2, 13*, Gressor (ed). Academic Press: New York.

TAYLOR-PAPADIMITRIOU, J. & ROZENGURT, E. (1985). Interferons as regulators of cell growth and differentiation. In *Interferons: Their Impact on Biology and Medicine*, Taylor-Papadimitriou, J. (ed). Oxford University Press: Oxford.

TWENTYMAN, P.R., WORKMAN, P., WRIGHT, K.A. & BLEEHEN, N.M. (1985). The effects of $\alpha$ and $\gamma$ Interferons on human lung cancer cell growth in vitro or as xenografts in nude mice. *Br. J. Cancer*, **52**, 21.

WATSON, J.V. (1980). Enzyme kinetic studies in cell populations using fluorogenic substrates and flow cytometric techniques. *Cytometry*, **1**, 143.

WATSON, J.V., CHAMBERS, S.C., WORKMAN, P. & HORSNELL, T.S. (1977). A flow cytometric method for measuring enzyme reaction kinetics in populations of single cells. *FEBS Letts*, **81**, 179.